# Effects of Combined Far-Infrared Radiation and Acupuncture at ST36 on Peripheral Blood Perfusion and Autonomic Activities

**DOI:** 10.1155/2017/1947315

**Published:** 2017-08-13

**Authors:** Cheng-Chan Yang, Gen-Min Lin, Jen-Hung Wang, Hsiao-Chiang Chu, Hsien-Tsai Wu, Jian-Jung Chen, Cheuk-Kwan Sun

**Affiliations:** ^1^Department of Electrical Engineering, National Dong Hwa University, Hualien 97401, Taiwan; ^2^Department of Chinese Medicine, Buddhist Tzu Chi General Hospital, Hualien 97002, Taiwan; ^3^Department of Medicine, Hualien-Armed Forces General Hospital, Hualien 97144, Taiwan; ^4^Department of Medicine, Tri-Service General Hospital, National Defense Medical Center, Taipei 114, Taiwan; ^5^Department of Medical Research, Buddhist Tzu Chi General Hospital, Hualien 97002, Taiwan; ^6^Department of Chinese Medicine, Taichung Tzu Chi Hospital, Buddhist Tzu Chi Medical Foundation, Taichung 42743, Taiwan; ^7^School of Chinese Medicine, Tzu Chi University, Hualien 97002, Taiwan; ^8^Department of Emergency Medicine, E-Da Hospital, I-Shou University, Kaohsiung 82445, Taiwan

## Abstract

Using four-channel photoplethysmography (PPG) for acquiring peripheral arterial waveforms, this study investigated vascular and autonomic impacts of combined acupuncture-far infrared radiation (FIR) in improving peripheral circulation. Twenty healthy young adults aged 25.5 ± 4.6 were enrolled for 30-minute measurement. Each subject underwent four treatment strategies, including acupuncture at ST36 (Zusanli), pseudoacupuncture, FIR, and combined acupuncture-FIR at different time points. Response was assessed at 5-minute intervals. Area under arterial waveform at baseline was defined as Area_Baseline_, whereas Area_Stim_ referred to area at each 5-minute substage during and after treatment. Area_Stim_/Area_Baseline_ was compared at different stages and among different strategies. Autonomic activity at different stages was assessed using low-to-high frequency power ratio (LHR). The results demonstrated increased perfusion for each therapeutic strategy from stage 1 to stage 2 (all *p* < 0.02). Elevated perfusion was noted for all treatment strategies at stage 3 compared to stage 1 except pseudoacupuncture. Increased LHR was noted only in subjects undergoing pseudoacupuncture at stage 3 compared to stage 1 (*p* = 0.045). Reduced LHR at stage 2 compared to stage 1 was found only in combined treatment group (*p* = 0.041). In conclusion, the results support clinical benefits of combined acupuncture-FIR treatment in enhancing peripheral perfusion and parasympathetic activity.

## 1. Introduction

Far-infrared radiation (FIR), which comprises electromagnetic waves of wavelength 3–1000 *μ*m [1], possesses physiological actions because not only of its high penetrating power in human tissue but also of its ability to elicit both heat-related [2] and nonheat-related [3] biological effects. It has been demonstrated that FIR causes vasodilatation, thereby improving human tissue perfusion [2, 4, 5] and skin microperfusion in rats through enhancing the action of endothelial nitric oxide synthase, eNOS, in vascular endothelium [6]. FIR has also been shown to suppress vascular endothelial proliferation and enhance endothelial repair through suppressing the action of vascular endothelial growth factor [4, 7, 8]. Besides, improvement of wound healing has also been reported through FIR-induced fibroblast recruitment and collagen disposition [9, 10]. Other demonstrated therapeutic actions of FIR also include the suppression of tumor cell proliferation and spreading [11–14], reduction of intravascular lipid deposition and risk of arteriosclerosis [15], and improving sleep quality [16].

On the other hand, acupuncture is a traditional Chinese therapeutic approach [17] that has been proposed to exert its actions through neurovascular modulation [18]. It has been accepted as a valid therapeutic option by the World Health Organization (WHO) which listed 64 indications for the procedure including neurological and vascular diseases such as seizure, headache, Parkinson's disease, and stroke [3]. The present study aimed at investigating whether improved therapeutic benefits are achievable through a combination of invasive acupuncture and noninvasive FIR by using the noninvasive tools of photoplethysmography (PPG) and heart rate variability for the assessment of peripheral perfusion and autonomic nervous activities, respectively.

## 2. Materials and Methods

### 2.1. Subject Population

Twenty healthy young adult volunteers (17 males and 3 females) were recruited. Individuals with known systemic diseases as well as habits of smoking and drinking were excluded from this study. All participants were required to have breakfast but refrained from beverages with alcohol or caffeine within 24 hours of the examinations. Besides, basic demographic (i.e., age and gender), anthropometric (i.e., body weight, body height, and body-mass index), and hemodynamic (i.e., systolic and diastolic pressure) information was recorded from all testing subjects. No acupuncture was performed on all 20 testing subjects one month prior to the present study. The study protocol was reviewed and approved by the Institutional Review Board on Ethics of Buddhist Tzu Chi General Hospital (IRB number IRB103-152-A).

### 2.2. Equipment and Data Acquisition

Four-channel photoplethysmography (PPG) (Lite-On Electronics Co., Tianjin, China) and electrocardiography (ECG) (Clamp Electrode 0102014, Qingdao Bright Medical Manufacturing Co., Ltd., China) were used for acquisition of data on pulse volume and heart rate variability (HRV), respectively. Signals of blood flow from PPG were filtered with second-order high-pass filter and low-pass filter with cut-off frequencies of 0.48-10 Hz, while signals from Lead II of ECG went through notch filter (59–61 Hz) and band-pass filter (0.98–19.4 Hz) before being processed with a analog-to-digital converter (USB-6210 DAQ, National Instruments, TX, USA) with a sampling rate of 500 Hz to 16-bit digital signals which were then stored in a computer with appropriate software (Labview Signal Express 2012, National Instruments, TX, USA) for waveform monitoring and analysis [11]. WS TY-101N emitter (Far IR Medical Technology Co., Ltd., Taipei, Taiwan) was used for sessions 3 and 4. The wavelength of the light generated from the electrified ceramic plates was in the range between 3 and 25 *μ*m with a peak at 5 *μ*m. Intensity is 20 mW/cm^2^ at 20 cm distance.

### 2.3. Procedures of Examinations

**Figure 1:**
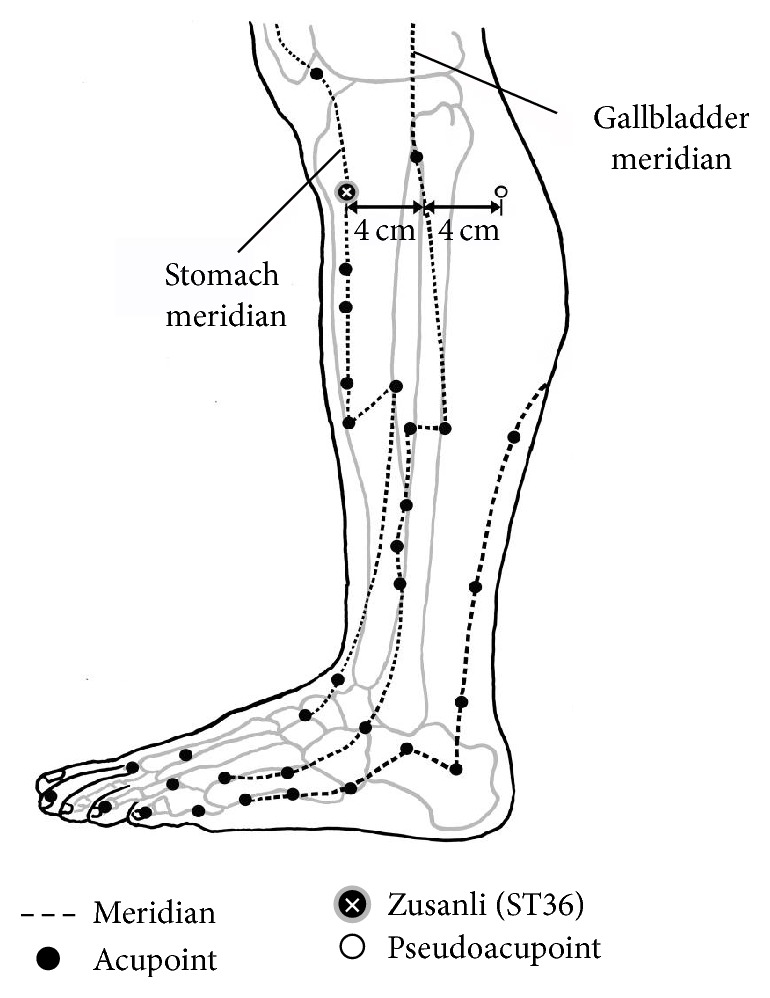
Locations of acupoints along the stomach and gallbladder meridians over the lower leg. Acupuncture performed at acupoint Zusanli (ST36) located 4 cm anterior to the gallbladder meridian. Pseudoacupoint chosen on the same horizontal plane 4 cm posterior to gallbladder meridian.

**Figure 2:**
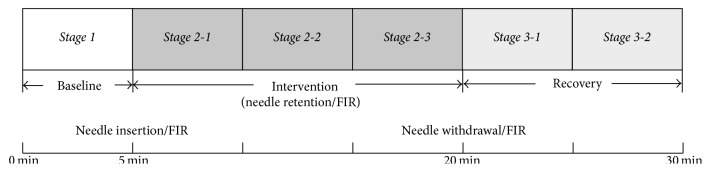
Recording lasting for 30 minutes with the first 5 minutes being the baseline (stage 1), 5th to 20th minute being intervention phase (stage 2), and 20th to 30th minute being the recovery phase (stage 3). FIR: far-infrared radiation.

Testing subjects were allowed to rest in supine position for over 5 minutes before data acquisition during which 4 PPG detectors were, respectively, attached to bilateral index finger as well as the second toe, while the three ECG detectors were, respectively, attached to the right wrist and bilateral ankles. Each subject received four different interventions during the experimental sessions: Session 1 (acupuncture), Session 2 (pseudoacupuncture), Session 3 (far-infrared radiation (FIR)), and Session 4 (acupuncture combined with FIR). During each session, the initial 5 minutes are defined as baseline stage (stage 1), the following 15 minutes as intervention stage (stage 2), and the last 10 minutes as postintervention (i.e., recovery) stage (stage 3). The second stage of sessions 1 and 4 involved the insertion of a 4 cm acupuncture needle at Zusanli following 5 minutes of baseline recording. The identification of Zusanli and the procedure of acupuncture were in accordance with the standard of traditional Chinese medical practice. With the knee flexed, the acupoint “Dubi” (ST-35) can be identified as the depression lateral to the patellar ligament. Zusanli (ST36) was located on horizontal plane 4 fingerbreadths below “Dubi” and is one fingerbreadth lateral to the anterior border of the tibia on vertical plane. The second stage of session 2 (pseudoacupuncture) involved the insertion of a 4 cm acupuncture needle at pseudoacupoint following 5 minutes of baseline recording. The pseudoacupoint was located on the same horizontal plane as ST36 at a point 8 cm posterior to ST36 so that ST36 and the pseudoacupoint were each 4 cm away from and on both sides of the gallbladder meridian (Figure 1). The pseudoacupoint was chosen because it was not located on any meridian to ensure the absence of acupunctural effect. The needle was inserted perpendicularly through the skin with a twisting motion of the thumb and index finger with repeated clockwise and anticlockwise rotation of the needle between 90 to 180 degrees till the state of* “Deqi”* (i.e., a numbness sensation signifying correct position of the needle) was achieved. The depth of penetration was the full length (4 cm) of a 32 G acupuncture needle (Toba acupuncture needle, Seoul, South Korea). The needle was allowed to stay in place for 15 minutes before being removed. An experienced physician of traditional Chinese medicine was responsible for acupuncture in all testing subjects. During the first stage of data acquisition, six sets of 5-minute signals were obtained. At stage 2 of the study, data were acquired for 15 minutes after placing the needle in the appropriate position through confirming the tingling and mild soreness sensation of the punctured site (i.e.,* Deqi*) with the testing subject. The recording then continued for 10 more minutes after the needle was withdrawn. Therefore, data acquisition lasted for totally 30 minutes (Figure 2).

### 2.4. Assessment of Peripheral Blood Flow and Autonomic Nervous Activities

Physiological signals before, during, and after acupuncture were recorded, including area under arterial waveforms (i.e., volume of blood flow) and electrocardiogram from which the time periods between two successive R waves (i.e., R-R intervals, RRI) were obtained and processed with Fast Fourier Transform (FFT) for determination of heart rate variability (HRV). The computation then yielded low frequency (LF) power, high frequency (HF) power, and low-to-high frequency ratio (LHR). The areas under the arterial waveforms within 5 minutes of baseline recording were summated (Area_Baseline_). The areas under the waveforms during 15 minutes of intervention (i.e., acupuncture or FIR) and 10 minutes postintervention (i.e., recovery phase) were divided into 5 parts each of which represented 5 minutes of recordings (Figure 2). Area_Stim_ is defined as the area under the waveforms for each five-minute recording at each substage after baseline. The change in blood flow every 5 minutes after intervention compared with the baseline was then expressed as a fraction: Area_Stim_/Area_Baseline_. Therefore, five values were obtained from each testing subject after baseline recording.

On the other hand, the present study also noninvasively assessed the changes in autonomic nervous activities through electrocardiograms by investigating the variations in RRI which, after Fast Fourier Transform (FFT), reflects the HRV. The power spectrum (0–0.04 Hz) after FFT can be divided into 4 parts, namely, ultralow frequency power (0–0.003 Hz), very low frequency power (0.003–0.04 Hz), low frequency power (0.04–0.15 Hz), and high frequency power (0.15–0.4 Hz). While the low frequency power (LFP) reflects sympathetic activities, the high frequency power (HFP) represents parasympathetic activities. The activity of the sympathetic nervous system relative to that of the parasympathetic can then be given by the LFP/HFP ratio (LHR).

### 2.5. Statistical Analysis

Data are expressed as mean ± SD. All statistical analyses were performed using SPSS software version 17.0 (SPSS Inc., Chicago, IL, USA). Repeated measures ANOVA was used for determining the significance of fluctuation throughout the course of treatment for each therapeutic strategy, while paired *t*-test was adopted for analyzing the significance of difference in measurement parameters for the same testing subject at different stages of treatment. A *p* value < 0.05 was considered statistically significant.

## 3. Results

### 3.1. Characteristics of Testing Subjects

The mean age of the 20 volunteers (17 males and 3 females) was 25.5 ± 4.6 (range, 19–37). The mean body-mass index was 24.2 ± 4.0 (range, 17.6–32.0). The mean systolic and diastolic pressure was 124.2 ± 14.5 mmHg (range, 100–163 mmHg) and 75.3 ± 10.6 mmHg (range, 62–98 mmHg), respectively. The mean heart rate was 76.1 ± 10.2 beats per minute (range, 63–106).

### 3.2. Changes in Peripheral Perfusion

**Figure 3:**
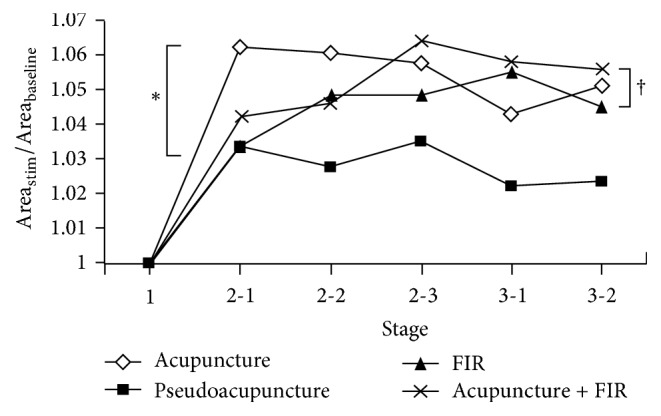
Changes in peripheral perfusion in testing subjects (*n* = 20) on receiving four different treatment strategies. Area_Baseline_: summation of areas under arterial waveforms within 5 minutes of baseline recording. Area_Stim_: summation of areas under arterial waveforms for each five-minute recording at each substage after baseline. FIR: far-infrared radiation. ^*∗*^*p* < 0.02 at stage 2 versus stage 1 for each treatment strategy; ^†^*p* < 0.04 at stage 3 versus Stage 1 for acupuncture, FIR, and combined treatment.

As shown in Figure 3, all subjects showed a significant increase in perfusion for each therapeutic strategy on entering from stage 1 to stage 2 (all *p* < 0.02) (Figure 3, Table 1). On the other hand, significant elevation in perfusion was noted for all treatment strategies at stage 3 compared to that at stage 1 with the exception of pseudoacupuncture. Considering the overall differences among all treatment strategies at stage 2 and stage 3, no remarkable difference in perfusion was noted at both stages.

### 3.3. Changes in LFP/HFP Ratio (LHR)

**Figure 4:**
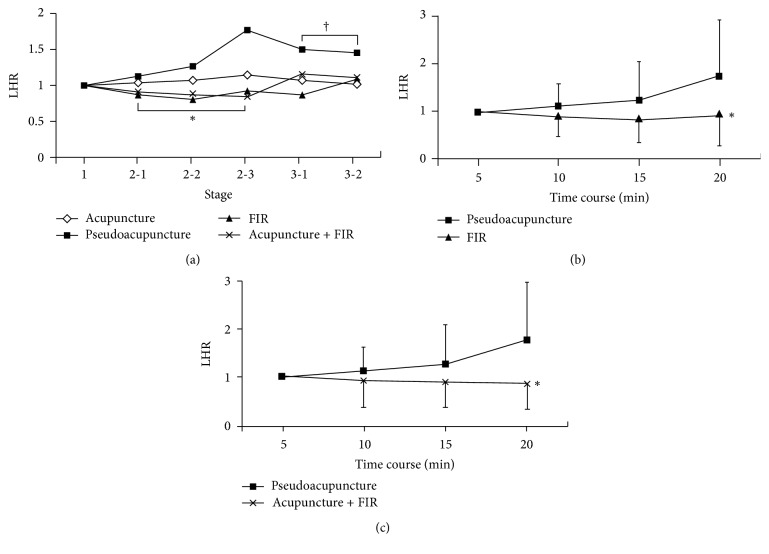
(a) Changes in LHR at different stages of treatment for subjects undergoing four kinds of therapies. LHR: low frequency power to high frequency power ratio; FIR: far-infrared radiation. ^*∗*^*p* = 0.041 at stage 2 versus stage 1 for subjects receiving acupuncture-FIR combined treatment; ^†^*p* = 0.045 at stage 3 versus stage 1 for subjects undergoing pseudoacupuncture. Significance of difference determined by paired *t*-test. (b) Comparison of changes in LHR between pseudoacupuncture and acupuncture treatment. ^*∗*^*p* = 0.012 determined with post hoc test; (c) comparison of changes in LHR between pseudoacupuncture and combined acupuncture-FIR treatment. ^†^*p* = 0.019 determined with post hoc test.

In terms of the impact of treatment strategy on LHR, significant increase in LHR was noted only in subjects undergoing pseudoacupuncture at stage 3 compared to that at stage 1 (*p* = 0.045) but not in the same subjects undertaking other treatment strategies (Figure 4(a), Table 1). Notable drop in LHR at stage 2 compared to that at stage 1 was demonstrated only in subjects receiving combined treatment (*p* = 0.041). At stage 2, subjects undertaking pseudoacupuncture showed significantly higher LHR (*p* = 0.013) compared to that when they received FIR (Figure 4(b)) or combined treatment (Figure 4(c)). Both time course and choice of therapeutic strategy had no significant impact on LHR at stage 3.

## 4. Discussion

The current study represents the first investigation into the therapeutic benefits of a combination of two alternative medical approaches to enhancing neurovascular activities including peripheral perfusion (i.e., increase in PI) and parasympathetic tone (i.e., decrease in LHR). The results showed that FIR, acupuncture, and the combination of both could cause an increase in perfusion that persisted through stage 2 (i.e., intervention period) and stage 3 (i.e., recovery period) of treatment, despite the lack of significant difference among the four treatment strategies. On the other hand, significant drop in LHR, which represents an increase in parasympathetic activity, was noted only in subjects receiving combined treatment at stage 2 (*p* = 0.041). Although both acupuncture and combined acupuncture-FIR groups showed a decrease in LHR from stage 1 to stage 2, the reduction failed to reach statistical significance. On the contrary, the pseudoacupuncture group demonstrated an increase in LHR on entering stage 2 in spite of being insignificant. The elevation in LHR, which signifies decreased parasympathetic activity, was noted in the subjects undergoing pseudoacupuncture at stage 3 of treatment (*p* = 0.045). The increase in LHR was probably due to pain-induced stimulation of sympathetic tone. The findings, therefore, support the clinical benefits of adopting acupuncture and FIR as both monotherapeutic regimen and combined treatment in terms of augmenting peripheral perfusion and parasympathetic activity.

A number of experimental and clinical studies have shown that acupuncture enhances the generation of nitric oxide (NO) and increases local circulation [19–21]. Several theories have been proposed to explain the phenomenon, including reduction of neuronal apoptosis [22–24], augmentation of antioxidative activity [25–27], and enhancement of neurotransmission [28–30]. A previous study in a rat model showed that acupuncture at ST36 can trigger the generation of nitric oxide in the gracile nucleus that modulates the control of blood pressure and heart rate [31]. Besides, acupuncture at ST36 has been found to boost intestinal motility in rats by elevating parasympathetic tone through vagus nerve stimulation [32]. Consistently, it has been reported that ST36 acupuncture in a rat model can enhance parasympathetic but suppress sympathetic activities [33]. Clinically, a study on the application of transcutaneous electrical nerve stimulation (TENS) at ST36 in patients with scleroderma demonstrated an alleviation of gastrointestinal symptoms through achieving sympathovagal balance [34]. Another clinical study applying TENS at ST36 in patients with functional dyspepsia also showed improved dyspeptic symptoms and increased high frequency heart rate variability possibly related to an increase in plasma neuropeptide Y level [35]. Furthermore, a previous study in healthy subjects revealed an increase in skin and muscle blood flow up to 50% after acupuncture at ST36 for 20 minutes [21].

Consistently, the current study showed that acupuncture could boost peripheral perfusion as previously reported [36, 37]. Nevertheless, combination of acupuncture and FIR had no additional benefit in terms of increasing peripheral blood flow in this study.

Similar to the vasodilatory action of acupuncture, not only has FIR been found to exhibit heat-related effect that involves resonance in tissue, but it has also been reported to trigger nonheat-related biochemical effects. Since human body consists of close to 70% of water, FIR-induced resonance of water molecules leads to severance of the hydrogen bonds, causing a cascade of thermally induced reactions [2]. Besides, using a FIR transmission model, it has been demonstrated that the energy can be absorbed by protein molecules of organisms to produce energy of quantity about that from hydrolysis of adenosine triphosphate [3]. The energy produced is transmitted among large molecules in an organism without raising the temperature or altering molecular structures, accounting for the nonheat-related biological effect which is the predominant contributor to FIR-related physiological action. The results of the present study demonstrated that FIR could increase peripheral perfusion. On the other hand, significant elevation in parasympathetic activity was noted only when combining FIR and acupuncture. One of the interesting findings in the present study is the persistence of perfusion enhancement effect during the recovery phase (i.e., stage 3) in subjects receiving acupuncture, FIR, or combined treatment. The findings, therefore, are consistent with the previously reported carryover effects of acupuncture [38] and FIR [6]. Nevertheless, further enhancement in perfusion during the recovery phase after FIR treatment described in a previous animal study [6] was not noted in the current investigation.

Heart rate variability (HRV), which is an easily available physiological parameter reflecting autonomic neural activities, has been widely used in medical research [39]. While low frequency (LF) power represents sympathetic tone, high frequency (HF) power reflects parasympathetic activity [40]. Since sympathetic and parasympathetic activities are antagonistic, LHR signifies the net sympathetic activity relative to that of the parasympathetic system. Chronic elevation in sympathetic tone has been reported to be a detrimental factor that contributes to the development of cardiovascular diseases such as hypertension [41], coronary artery disease [42], and stroke [43]. The present study demonstrated significant reduction in LHR after combined acupuncture-FIR treatment, highlighting the beneficial role of combining the two alternative therapies in neuromodulation. Moreover, the elevation in LHR after pseudoacupuncture further illustrates the positive therapeutic influence of acupuncture on the enhancement of parasympathetic activity.

There are several limitations in the present study. First, the relatively small number of testing subjects precluded the drawing of a strong conclusion from the findings. On the other hand, the same subject was enrolled to undergo different kinds of therapy with an interval of at least one week between two strategies not only to eliminate individual variation but also to minimize the residual effect from previous treatment. Second, since the real-world clinical practice involves several sessions of acupuncture and FIR to achieve the anticipated outcomes, the utilization of acupuncture and FIR only once in the present study may have underestimated the therapeutic effects of the two approaches both as monotherapy or as combined treatment. Third, only healthy young adults were enrolled in this study so that the impact of age and disease on treatment outcomes was not evaluated.

In conclusion, the present study demonstrated the clinical benefits of utilizing acupuncture and FIR as both monotherapeutic strategies and combined treatment in terms of enhancing peripheral perfusion and parasympathetic activity, underscoring the positive roles of the two alternative therapies in neurovascular modulation.

## Figures and Tables

**Table 1:** Changes in peripheral perfusion and LHR for four different treatments at different stages in the same group of testing subjects (*n* = 20).

Parameter	Treatment	Stage 1	Stage 2	^*∗*^ *p* value^a^	Stage 3	^*∗*^ *p* value^b^
Perfusion (aU)	Acupuncture	2.20 ± 0.35	2.33 ± 0.36	<0.001	2.29 ± 0.30	0.001
	Pseudoacupuncture	2.33 ± 0.30	2.41 ± 0.29	<0.001	2.38 ± 0.26	0.069
	FIR	2.30 ± 0.31	2.40 ± 0.35	0.020	2.42 ± 0.37	0.032
	Acupuncture-FIR	2.26 ± 0.45	2.37 ± 0.44	<0.001	2.38 ± 0.41	0.004

LHR	Acupuncture	1.84 ± 1.14	1.64 ± 0.83	0.442	1.62 ± 0.80	0.306
	Pseudoacupuncture	1.58 ± 0.89	1.80 ± 0.85	0.206	2.00 ± 1.17	0.045
	FIR	2.07 ± 0.97	1.72 ± 0.88	0.108	2.07 ± 1.89	0.601
	Acupuncture-FIR	2.42 ± 1.58	1.74 ± 0.93	0.041	2.15 ± 1.48	0.691

aU: arbitrary unit; FIR: far-infrared radiation; LHR: low frequency power to high frequency power ratio. ^a^Comparison between stages 1 and 2. ^b^Comparison between stages 3 and 1. ^*∗*^Significance of difference determined by paired *t*-test.
